# Medium-Chain Fatty Acid Synthesis by “*Candidatus* Weimeria bifida” gen. nov., sp. nov., and “*Candidatus* Pseudoramibacter fermentans” sp. nov.

**DOI:** 10.1128/AEM.02242-19

**Published:** 2020-01-21

**Authors:** Matthew J. Scarborough, Kevin S. Myers, Timothy J. Donohue, Daniel R. Noguera

**Affiliations:** a The Great Lakes Bioenergy Research Center, University of Wisconsin—Madison, Madison, Wisconsin, USA; b The Department of Civil and Environmental Engineering, University of Wisconsin—Madison, Madison, Wisconsin, USA; c The Department of Bacteriology, University of Wisconsin—Madison, Madison, Wisconsin, USA; University of Bayreuth

**Keywords:** Ech complex, *Firmicutes*, MCFA, *Pseudoramibacter*, RNF complex, “*Ca*. Weimeria,” chain elongation, hydrogenase

## Abstract

Chain elongation is emerging as a bioprocess to produce valuable medium-chain fatty acids (MCFA; 6 to 8 carbons in length) from organic waste streams by harnessing the metabolism of anaerobic microbiomes. Although our understanding of chain elongation physiology is still evolving, the reverse β-oxidation pathway has been identified as a key metabolic function to elongate the intermediate products of fermentation to MCFA. Here, we describe two uncultured chain-elongating microorganisms that were enriched in an anaerobic microbiome transforming the residues from a lignocellulosic biorefining process. Based on a multi-omic analysis, we describe “*Candidatus* Weimeria bifida” gen. nov., sp. nov., and “*Candidatus* Pseudoramibacter fermentans” sp. nov., both predicted to produce MCFA but using different substrates. The analysis of a time series metatranscriptomic data set suggests that “*Ca*. Weimeria bifida” is an effective xylose utilizer since both the pentose phosphate pathway and the bifid shunt are active. Furthermore, the metatranscriptomic data suggest that energy conservation during MCFA production in this organism is essential and occurs via the creation of an ion motive force using both the RNF complex and an energy-conserving hydrogenase. For “*Ca*. Pseudoramibacter fermentans,” predicted to produce MCFA from lactate, the metatranscriptomic analysis reveals the activity of an electron-confurcating lactate dehydrogenase, energy conservation via the RNF complex, H_2_ production for redox balance, and glycerol utilization. A thermodynamic analysis also suggests the possibility of glycerol being a substrate for MCFA production by “*Ca*. Pseudoramibacter fermentans.” In total, this work reveals unknown characteristics of MCFA production in two novel organisms.

**IMPORTANCE** Chain elongation by medium-chain fatty acid (MCFA)-producing microbiomes offers an opportunity to produce valuable chemicals from organic streams that would otherwise be considered waste. However, the physiology and energetics of chain elongation are only beginning to be studied, and many of these organisms remain uncultured. We analyzed MCFA production by two uncultured organisms that were identified as the main MCFA producers in a microbial community enriched from an anaerobic digester; this characterization, which is based on meta-multi-omic analysis, complements the knowledge that has been acquired from pure-culture studies. The analysis revealed previously unreported features of the metabolism of MCFA-producing organisms.

## Abstract (precis)

Chain elongation by medium-chain fatty acid (MCFA)-producing microbiomes offers an opportunity to produce valuable chemicals from organic streams that would otherwise be considered waste. However, the physiology and energetics of chain elongation are only beginning to be studied, and many of these organisms remain uncultured. We analyzed MCFA production by two uncultured organisms that were identified as the main MCFA producers in a microbial community enriched from an anaerobic digester; this characterization, which is based on meta-multi-omic analysis, complements the knowledge that has been acquired from pure-culture studies. The analysis revealed previously unreported features of the metabolism of MCFA-producing organisms.

## INTRODUCTION

Chain elongation has been proposed as a microbial process to produce valuable chemicals from complex waste feedstocks (1). This bioprocess relies on the combined metabolism of an anaerobic microbiome to hydrolyze complex organic substrates, ferment the hydrolyzed products to small organic intermediates (2 to 3 carbon molecules), and elongate these fermentation products to medium-chain fatty acids (MCFA; 6 to 8 carbon molecules) through reverse β-oxidation (1). MCFA are an attractive product due to their high value, relatively low solubility in water, and potential to offset fossil fuel demands for petrochemicals and other products (2, 3). Bioreactors performing chain elongation also provide model systems for studying the metabolic contributions of uncultured organisms to this process. To date, all but one of the known chain-elongating bacteria belong to the *Clostridia* class within the *Firmicutes* phylum (1). These chain-elongating bacteria primarily use lactate (4, 5), ethanol (6), or carbohydrates (7) to drive MCFA production.

We recently described a chain-elongating microbiome that produced sufficient hexanoic and octanoic acids from lignocellulosic biorefining residues to reduce the minimum selling price of ethanol produced in a biorefinery (2). Using a combination of metagenomics and metatranscriptomics, we characterized this microbiome as having a small set of high-abundance organisms (8), with two populations within the *Clostridia* class performing chain elongation. One high-abundance member of this microbiome (LCO1) (8) belonged to the *Lachnospiraceae* family and was predicted to produce MCFA from xylose and other pentoses; the second one (EUB1) (8) corresponded to the *Eubacteriaceae* family and was predicted to produce MCFA from lactate.

Here, we combined multi-omic approaches to further analyze the genomic and metabolic features of these two predicted MCFA-producing organisms, which remain uncultured. A time series gene expression analysis showed that transcripts encoding proteins predicted to be involved in reverse β-oxidation are among the most abundant transcripts after the lignocellulosic biorefinery residues are fed to the microbial community. Our analysis also reveals that both organisms contain transcripts that encode a proton-translocating energy conserving hydrogenase, suggesting contributions of previously unreported metabolic networks to MCFA production. Based on these new results, we conclude that LCO1 represents a novel genus within the *Lachnospiraceae* family and propose the name of “*Candidatus* Weimeria bifida” gen. nov., sp. nov. Our data also predict that EUB1 represents a new species within the *Pseudoramibacter* genus, and we propose the name “*Candidatus* Pseudoramibacter fermentans” sp. nov. to represent this new species. In total, the objective of this work is to provide a greater understanding of MCFA-producing organisms in the context of a chain-elongating microbiome.

## RESULTS AND DISCUSSION

### Refinement of MAGs.

We previously reported the construction of draft metagenome-assembled genomes (MAGs) from an MCFA-producing microbiome fed with lignocellulosic biorefinery residues, in which LCO1 and EUB1 represented the abundant chain-elongating microorganisms (8). These draft MAGs were constructed using DNA samples from the first 120 days of reactor operation. To improve the quality of these MAGs, we obtained Illumina and PacBio sequencing reads from the same microbiome at additional times during a 378-day operational period. We coassembled 244 million Illumina Hi-seq (2 by 250 bp) reads from five time points (days 96, 120, 168, 252, and 378) (see Fig. S1 in the supplemental material) into 24,000 contigs. Contigs were binned into MAGs; the MAGs with relative abundance greater than 1% were then gap filled with PacBio reads from the day 378 sample. This analysis resulted in an overall improvement in MAG quality with respect to completeness, contamination, and number of scaffolds (Table S1). The two most abundant MAGs derived from this analysis (Table 1), LCO1.1 and EUB1.1 (accounting for ∼80% of the recovered DNA sequences) (Data File S1), provide improved predictions for the genetic makeup of the organisms previously denoted as LCO1 and EUB1 (8), respectively.

**TABLE 1:** Summary of metagenome-assembled genomes from an MCFA-producing bioreactor

Organism	Previous name^*a*^	Relative abundance (%)^*b*^	Completeness (%)^*c*^	Contamination (%)^*c*^	Genome size (Mbp)^*c*^	No. of scaffolds^*c*^
“*Ca*. Weimeria bifida”	LCO1	75.3	96.9 (95.4)	0.5 (0.0)	2.39 (2.10)	10 (44)
“*Ca*. Pseudoramibacter fermentans”	EUB1	4.7	99.2 (97.8)	0.2 (0.2)	2.29 (2.00)	29 (35)

a Name reported in Scarborough et al. (8).

b Relative abundance based on DNA read mapping normalized to genome size for the day 252 sample. This date corresponds to the date the metatranscriptomic experiment was initiated.

c Numbers in parentheses indicate values for metagenome-assembled genomes reported in Scarborough et al. (8).

### Phylogenetic classification of LCO1.1 and EUB1.1.

**FIG 1:**
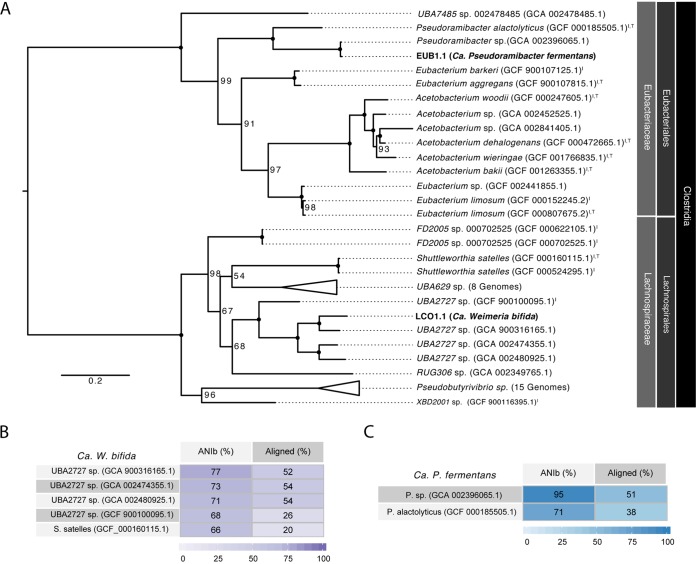
Phylogenetic analysis of two metagenome-assembled genomes (shown in bold text) predicted to perform chain elongation in an anaerobic microbiome converting lignocellulosic residues to medium-chain fatty acids. (A) A phylogenetic tree was created based on concatenated amino acid sequences for 120 single-copy marker genes from the GTDB using RAxML. Bootstrap support values are indicated at the nodes. Closed circles indicate bootstrap support values of 100. Organisms that have representative isolates are indicated by an “I” superscript, and organisms representing NCBI type strains are indicated with a “T” superscript. Genome identifiers are provided in parentheses. (B) BLAST average nucleotide identity (ANIb) comparison for LCO1.1 (“*Ca*. Weimeria bifida”) to related genomes. (C) ANIb comparison for EUB1.1 (“*Ca*. Pseudoramibacter fermentans”) to related genomes.

The Genome Taxonomy Database (GTDB) tool kit (9) was used to provide a taxonomic classification of LCO1.1 and EUB1.1 (Fig. 1). The reconstructed LCO1.1 genome, which accounted for ∼75% of the mapped DNA reads (Table 1), clusters with a group of genomes identified in the GTDB with the name UBA2727 within the *Lachnospiraceae* family, in the order *Clostridia*, phylum *Firmicutes*. This cluster contains three MAGs obtained from rumen samples (GTDB accession no. GCA_002474355.1, GCA_002480925.1, and GCA_900316165.1) (9), as well as a genome of an isolate from the Hungate 1000 project (named *Lachnospiraceae* bacterium C10 in the NCBI database; GTDB accession no. GCF_900100095.1) (10) (Data File S2). A comparative analysis of LCO1.1 with the four representatives of the UBA2727 cluster (Fig. 1B) shows average nucleotide identity (ANI) values greater than 70% with the three MAGs and 68% with the Hungate 1000 project isolate. The most closely related type strain is Shuttleworthia satelles, an organism isolated from the human oral cavity and described as a carbohydrate consumer and butyrate producer (11). Given the low ANI value (66%) obtained when the LCO1.1 genome is compared to that of *S. satelles*, we propose here the name “*Candidatus* Weimeria” to define the organisms comprised of the UBA2727 cluster plus the LCO1.1 MAG. Further, we propose the epithet “bifida” to describe a species within this new genus, represented by LCO1.1.

The EUB1.1 MAG, which accounted for ∼5% of the mapped DNA reads (Table 1), clustered in the *Pseudoramibacter* genus, within the *Eubacteriaceae* family, also in the order *Clostridia*. This genus currently contains only one known species, defined by the isolate Pseudoramibacter alactolyticus (originally named Ramibacterium alactolyticum [12]), which was recovered from a human lung abscess and has been shown to produce short- and medium-chain fatty acids (12). The ANI of EUB1.1 to *P. alactolyticus* is 71%, with an alignment factor of 38% (Fig. 1C). The GTDB also contained a related MAG that was obtained from a sheep rumen metagenomic analysis (9) and identified as *Pseudoramibacter* sp. (GCA_002396065.1). This MAG had an ANI of 95% and an alignment factor of 51% to EUB1.1 (Fig. 1C). Its completion was estimated to be only 57%, which likely contributes to its low alignment factor to EUB1.1. A comparative analysis of these three *Pseudoramibacter* genomes (Data File S3) shows that among their similarities, they all contain genes typically associated with reverse β-oxidation, consistent with their predicted ability to produce MCFA. Based on the clustering of EUB1.1 within the *Pseudoramibacter* genus, on the obtained ANI values, and on the comparative analysis that follows, we propose here a new species within the *Pseudoramibacter* genus, represented by EUB1.1 and *Pseudoramibacter* sp., and identified with the epithet “fermentans.”

### Multi-omic analysis of “*Ca*. Weimeria bifida” and “*Ca*. Pseudoramibacter fermentans.”

Previous analyses showed that “*Ca*. Weimeria bifida” and “*Ca*. Pseudoramibacter fermentans” expressed genes related to MCFA production in the bioreactor at a single time point collected after 96 days of bioreactor operation (8). To further evaluate MCFA production by “*Ca*. Weimeria bifida” and “*Ca*. Pseudoramibacter fermentans,” we analyzed transcript abundance for 36 h after the bioreactor received a transient load of lignocellulosic biorefinery residue (Fig. S1 and Data Files S4 and S5). This experiment was performed after the reactor had been operating continuously for 252 days. Concurrent analysis of the medium over this time period showed that xylose and glycerol were consumed whereas lactate transiently accumulated in the reactor (Fig. S2). We used this multi-omic analysis to investigate substrate utilization, the enzymes involved in converting substrates into intermediates and end products, the potential for MCFA production via the reverse β-oxidation pathway, and the predicted energy-conserving features of the predominant MAGs in this anaerobic microbiome.

### (i) Chain elongation in “*Ca*. Weimeria bifida.”

**FIG 2:**
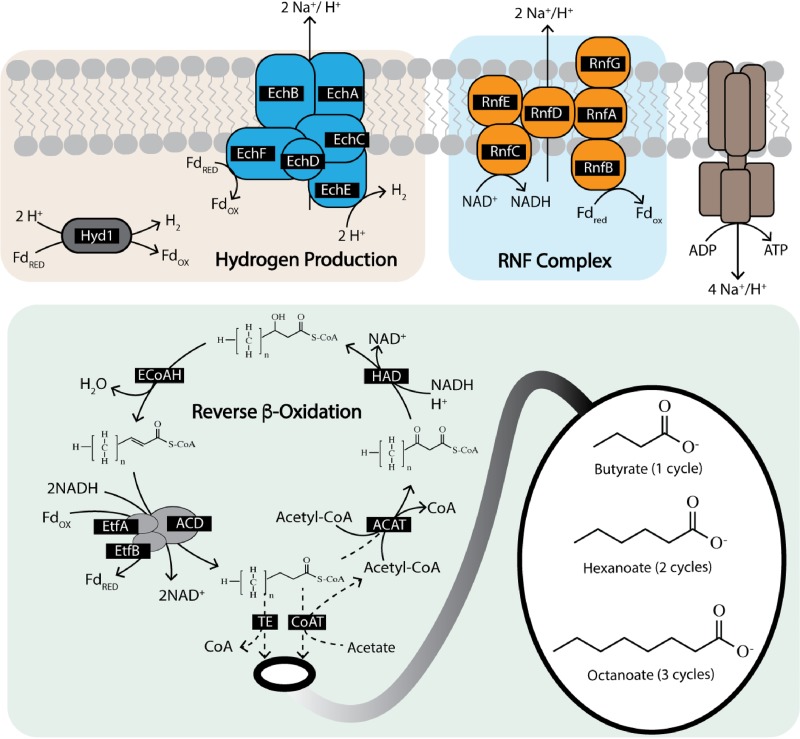
Metabolic pathways involved in chain elongation. Reverse β-oxidation is a four-step process using acyl-CoA acetyltransferase (ACAT), 3-hydroxy-acyl-CoA dehydrogenase (HAD), enoyl-CoA dehydratase (ECoAH), and acyl-CoA dehydrogenase (ACD). The reduction of enoyl-CoA with NADH can be combined with the reduction of ferredoxin through an electron-bifurcating acyl-CoA dehydrogenase complex containing EtfA and EtfB. The terminal enzyme of reverse β-oxidation can be either a CoA transferase (CoAT) or thioesterase (TE). Reverse β-oxidation is coupled with proton-translocating enzymes to generate ATP with reduced ferredoxin. This figure is partly adapted from previous work (8).

**FIG 3:**
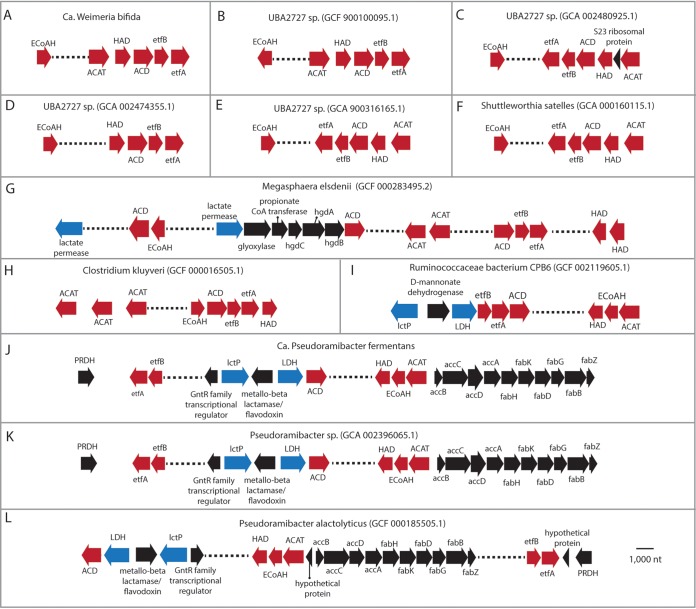
Organization of selected genes in organisms related to “*Ca*. Weimeria bifida,” “*Ca*. Pseudoramibacter fermentans,” and known chain-elongating organisms. Reverse β-oxidation genes are shown in red, and genes involved in lactate utilization, if present, are shown in blue. Reverse β-oxidation genes include acyl-CoA acetyltransferase (ACAT), 3-hydroxy-acyl-CoA dehydrogenase (HAD), enoyl-CoA dehydratase (ECoAH), acyl-CoA dehydrogenase (ACD), electron transfer flavoprotein A (*etfA*), and electron transfer flavoprotein B (*etfB*). Lactate utilization genes include lactate permease (*lctP*) and lactate dehydrogenase (LDH). Other genes included in the figure are prephenate dehydrogenase (PRDH) (J, K, and L), 2-hydroxyglutaryl-CoA dehydratase (*hgd*) involved in propionate production in Megasphaera elsdenii (G), and acetyl-CoA carboxylase (*acc*) and fatty acid biosynthesis (*fab*) genes when they are adjacent to reverse β-oxidation genes (J, K, and L). nt, nucleotides.

**FIG 4:**
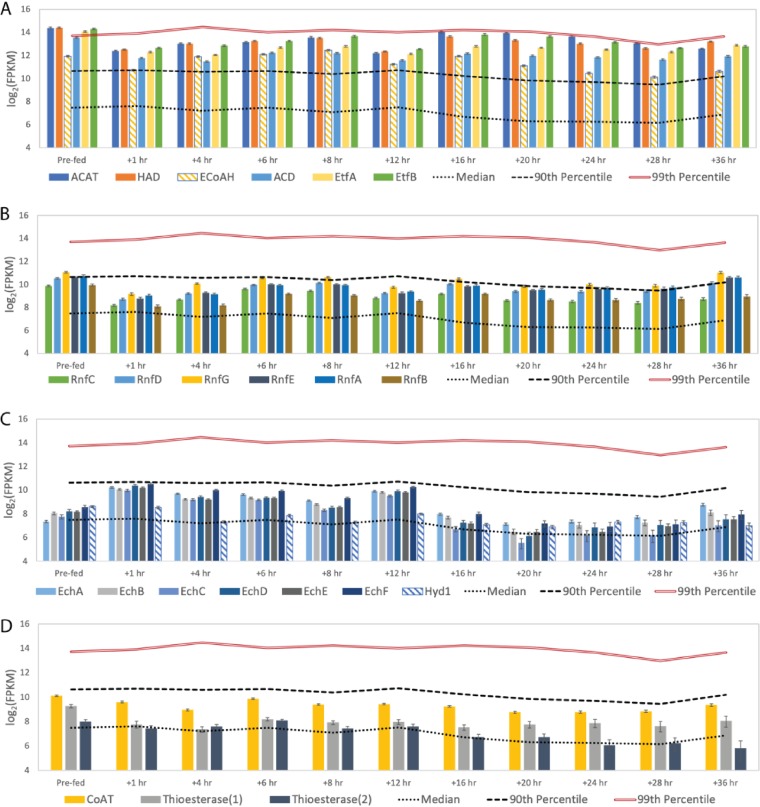
Transcript abundance for predicted chain elongation transcripts by “*Ca*. Weimeria bifida.” “Prefed” represents the time point prior to feeding the reactor. Error bars show 95% confidence levels determined with CuffLinks. Transcript abundance data are shown for transcripts encoding enzymes used for reverse β-oxidation (A), the RNF complex (B), hydrogenases (C), and terminal enzymes of reverse β-oxidation (D). The roles of the designated enzymes in chain elongation are provided in Fig. 2. FPKM, fragments per kilobase per million.

Four enzymes are known or predicted to comprise the reverse β-oxidation cycle (Fig. 2). In the first step, acyl-coenzyme A (CoA) acetyltransferase (ACAT) condenses an acetyl-CoA with an acyl-CoA; the product of this reaction is reduced by 3-hydroxy-acyl-CoA dehydrogenase (HAD), followed by dehydration by 2-enoyl-CoA with enoyl-CoA dehydratase (ECoAH) and reduction by an acyl-CoA dehydrogenase (ACD) to form an elongated acyl-CoA. In some organisms, the last dehydrogenation reaction is catalyzed by an electron-bifurcating energy-conserving enzyme whereby the enoyl-CoA reduction with NADH is paired with the reduction of ferredoxin through an ACD complex containing the electron transfer flavoproteins EtfA and EtfB (13). The “*Ca*. Weimeria bifida” genome has a gene cluster encoding ACAT, HAD, ACD, EtfA, and EtfB (Fig. 3A), while a gene predicted to encode ECoAH is located on a different region of the genome. The abundance of transcripts encoding reverse β-oxidation enzymes was at or above the 90th percentile in all samples analyzed (Fig. 4A). Pairwise comparisons of reverse β-oxidation transcript abundance show high correlations for genes in the cluster encoding ACAT, HAD, ACD, EtfA, and EtfB over the course of this analysis but low correlations with the ECoAH transcript (Fig. S3), suggesting that this later gene is not coexpressed with those predicted to be involved in the reverse β-oxidation pathway of “*Ca*. Weimeria bifida.”

Producing reduced ferredoxin during reverse β-oxidation constitutes a potential energy-conserving process (Fig. 2) because its subsequent oxidation can be used to create an ion motive force by either the RNF complex (RnfABCDEG), as proposed for the MCFA-producing Clostridium kluyveri (14), or the energy-conserving hydrogenase complex Ech, as described for Clostridium thermocellum (15). In “*Ca*. Weimeria bifida”, the genes encoding the subunits of the RNF complex are found in a single operon arranged as *rnfCDGEAB*, which is consistent with the gene organization in C. kluyveri, Clostridium difficile, and other *Clostridia* species (16). Genes encoding individual subunits of the Ech complex are also found in a single operon in “*Ca*. Weimeria bifida” (*echABCDEF*) (Data File S2) and are adjacent to genes encoding HypB, HypC, and HypD, which are involved in hydrogenase assembly (17, 18). Predicted *echABCDEF* gene clusters were not found in genomes of the other known chain-elongating organisms analyzed in this study (Data File S6), but they have been found in a diverse range of other bacteria and archaea (19).

Time series transcriptomic analysis showed that “*Ca*. Weimeria bifida” produced high levels of transcripts encoding the RNF and the Ech complexes when MCFA were produced after addition of lignocellulosic biorefinery residues (Fig. 4). Transcripts encoding subunits of the RNF complex (RnfABCDEFG) were present above the 90th percentile at several time points after addition of lignocellulosic biorefinery residues and found to be at or above median expression levels throughout the study (Fig. 4B). Transcripts for genes encoding subunits of the Ech hydrogenase complex followed a different pattern, with high-level abundance only for the first 12 h after addition of lignocellulosic biorefinery residues (Fig. 4C). We also found that transcripts of Ech complex genes were more abundant than those encoding a putative periplasmic ferredoxin hydrogenase (Hyd1), the only other hydrogenase predicted to be present in the “*Ca*. Weimeria bifida” genome. Thus, the multi-omic data support a role for both the RNF and Ech complexes during MCFA production, likely by conserving energy via generation of an ion motive force (Fig. 2).

The reverse β-oxidation cycle is also predicted to require either a CoA transferase (CoAT) or a thioesterase to remove the CoA from the terminal acyl-CoA molecule, thereby releasing the corresponding acid (Fig. 2). During the course of this experiment, “*Ca*. Weimeria bifida” expressed genes encoding one predicted CoAT and two predicted thioesterases. Transcripts of all three genes were at or above median levels throughout the time course of this experiment (Fig. 4D). The genes encoding putative thioesterase enzymes and CoAT are not located near other predicted reverse β-oxidation genes in the “*Ca*. Weimeria bifida” genome (Data File S2), but the abundance of transcripts encoding one of the predicted thioesterase enzymes (TE.1) (Fig. S3) is more closely correlated with other reverse β-oxidation pathway transcripts than the other predicted thioesterase (TE.2) (Fig. S3). TE.1 was annotated as a thioesterase superfamily protein (Data File S2), and a Basic Local Alignment Search Tool (BLAST) analysis of amino acid sequences (Data File S7) places it in the hotdog fold family of thioesterase proteins, some of which have been shown to cleave CoA from medium- and long-chain acyl-CoA molecules (20). Thus, this analysis indicates that CoAT and TE.1 may participate in the terminal step of reverse β-oxidation in “*Ca*. Weimeria bifida.”

### (ii) “*Ca*. Weimeria bifida” is predicted to use multiple routes to consume xylose as a source of carbon for MCFA production.

**FIG 5:**
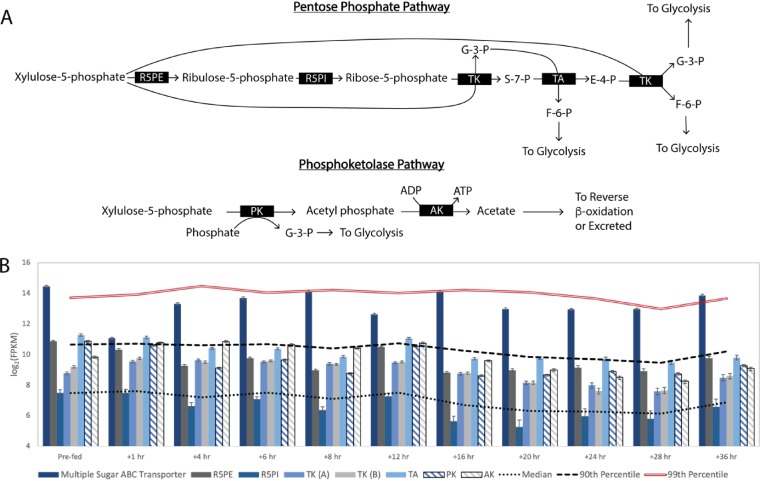
Predicted routes for xylose utilization by “*Ca*. Weimeria bifida.” (A) After xylose activation to xylulose-5-phosphate, “*Ca*. Weimeria bifida” can consume pentoses via the pentose phosphate pathway or the phosphoketolase pathway. Key enzymes involved in xylose consumption include ribulose 5-phosphate epimerase (R5PE), ribose-5-phosphate isomerase (R5PI), transketolase (TK), transaldolase (TA), transketolase (TK), phosphoketolase (PK), and acetate kinase (AK). (B) Transcript abundance for 36 h after providing the bioreactor with conversion residue. “Prefed” represents the time point prior to feeding the reactor. Error bars show 95% confidence levels determined with CuffLinks.

Previous studies (8) and the revised genome provided in this work predict that “*Ca*. Weimeria bifida” can metabolize xylose and other pentoses (Fig. 5A) as the main source of energy and carbon when grown in an MCFA-producing microbiome (8). In this time series experiment, we found that transcripts encoding an ABC transporter predicted to transport multiple sugars were present at or above the 99th percentile at several time points (Fig. 5B), suggesting a role for this protein in sugar uptake.

To investigate potential routes for sugar utilization by “*Ca*. Weimeria bifida,” we compared transcript abundances of genes encoding enzymes predicted to function in the pentose phosphate and phosphoketolase pathways after the addition of lignocellulosic biorefinery residues (Fig. 5B). Patterns of transcript abundance indicate that genes encoding enzymes for both pathways are expressed above median expression levels, suggesting that both pathways are used for pentose utilization and its subsequent conversion to intermediates that then enter the reverse β-oxidation pathway to produce MCFA.

### (iii) Predicted conservation of metabolic functions between “*Ca*. Weimeria bifida” and related organisms.

We found that all of the UBA2727 genomes, Shuttleworthia satelles, and the LCO1.1 genome contained genes needed for the reverse β-oxidation pathway (Data File S2). In the UBA2727, *S. satelles*, and “*Ca*. Weimeria bifida” genomes, the genes encoding ACAT, HAD, ACD, EtfA, and EtfB are clustered (Fig. 3A to F), whereas ECoAH is located on a different region of the genome. The presence of genes encoding each of these enzymes and their transcript abundance patterns in “*Ca*. Weimeria bifida” during MCFA production suggest that reverse β-oxidation and the electron-bifurcating ACD reaction are conserved and key features of chain elongation in each of these organisms. Genome comparisons revealed that the colocation of genes encoding ACD, EtfA, and EtfB is a common, although not essential, topology among known MCFA-producing organisms. Examples of other MCFA producers with this gene arrangement include Megasphaera elsdenii (Fig. 3G), C. kluyveri (Fig. 3H), and the bacterium *Ruminococcaceae* strain CPB6 (Fig. 3I).

While two routes of pentose utilization (the pentose phosphate and phosphoketolase pathways) are predicted to be present in the “*Ca*. Weimeria bifida” genome, none of the UBA2727 genomes nor the *S. satelles* genome contains a predicted phosphoketolase gene. This indicates that the use of multiple pentose consumption pathways may be a unique feature of “*Ca*. Weimeria bifida” not found in other members of the UBA2727 cluster or the nearest type strain. All of the UBA2727 genomes and the “*Ca*. Weimeria bifida” genome contain genes for acetate production, including phosphotransacetylase and acetate kinase, indicating that all of the species in this genus may produce acetate as a product of anaerobic sugar metabolism.

The putative phosphoketolase from “*Ca*. Weimeria bifida” is similar to enzymes predicted to be present in other firmicutes and betaproteobacteria, including three species of *Megasphaera* (Fig. S4), a genus containing known MCFA producers. The phosphoketolase pathway (Fig. 5A), termed the “bifid shunt” (21) in a *Bifidobacterium* (22), provides an alternative to glycolysis and the pentose phosphate pathway for sugar utilization. In this pathway, the phosphoketolase enzyme splits xylulose-5-phosphate into glyceraldehyde-3-phosphate and acetyl-phosphate (23). Energy can then be conserved through the phosphorylation of ADP and production of acetate by acetate kinase (Fig. 5A). Overall, the phosphoketolase pathway can produce more ATP than the pentose phosphate pathway per mole of xylose (Fig. S5), and it directs carbon to acetate in addition to producing glycolysis intermediates (Fig. 5A).

### (iv) Chain elongation by “*Ca*. Pseudoramibacter fermentans.”

**FIG 6:**
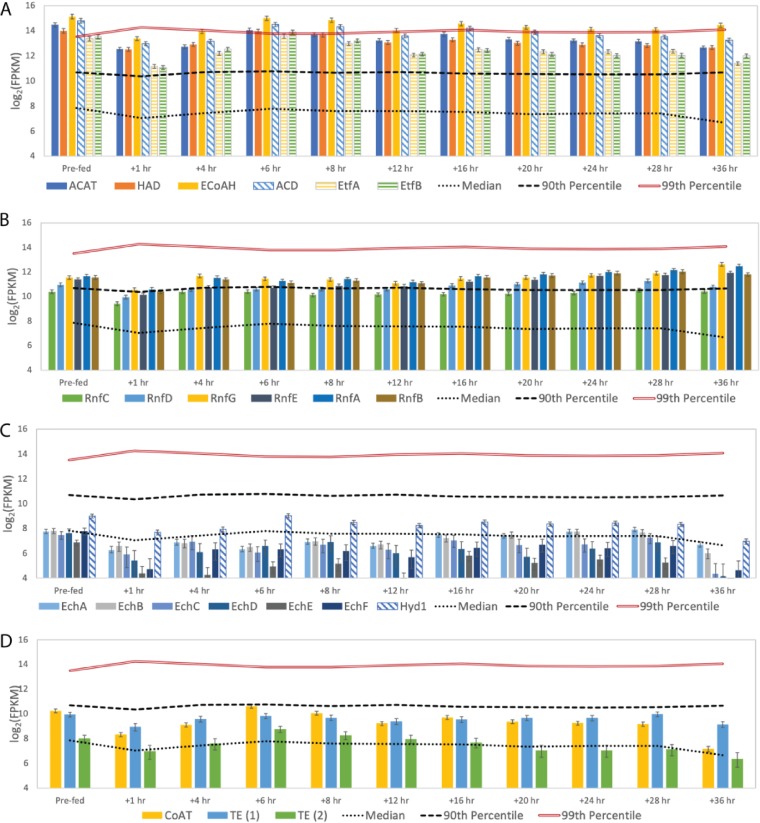
Transcript abundance for chain elongation transcripts by “*Ca*. Pseudoramibacter fermentans.” “Prefed” represents the time point prior to feeding the reactor. Error bars show 95% confidence levels determined with CuffLinks. Transcript abundance data are shown for genes encoding enzymes used for reverse β-oxidation (A), the RNF complex (B), hydrogenases (C), and terminal enzymes of reverse β-oxidation (D). The roles of enzymes in chain elongation and enzyme definitions are shown in Fig. 2.

**FIG 7:**
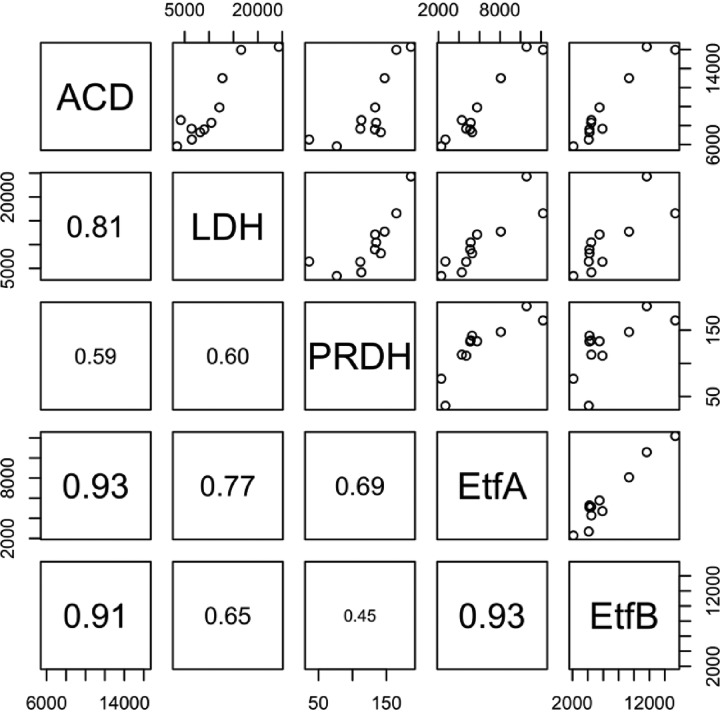
Pairwise linear correlation analyses for expression of “*Ca*. Pseudoramibacter fermentans” genes encoding lactate dehydrogenase (LDH), acetyl-CoA dehydrogenase (ACD), prephenate dehydrogenase (PRDH), electron transfer flavoprotein A (EtfA), and electron transfer flavoprotein B (EtfB). Values on the on *x* and *y* axes represent the number of fragments per kilobase per million. Numbers within the plots are coefficients of determination.

We previously predicted that “*Ca*. Pseudoramibacter fermentans” consumed lactate and produced MCFA when this microbiome was supplied with lignocellulosic biorefinery residues (8). In this study, we found that transcripts encoding enzymes predicted to function in the reverse β-oxidation pathway were among the most abundant in “*Ca*. Pseudoramibacter fermentans” after the addition of lignocellulosic biorefinery residues (Fig. 6A). However, in the revised “*Ca*. Pseudoramibacter fermentans” genome we found that the gene encoding the putative ECoAH protein is located near genes predicted to encode enzymes in the reverse β-oxidation cycle (Fig. 3), unlike the genomes of “*Ca*. Weimeria bifida” and related organisms. In addition, we found that the abundance of the transcript encoding the EcoAH protein correlates well with abundances of “*Ca*. Pseudoramibacter fermentans” genes predicted to encode other enzymes in the reverse β-oxidation cycle (Fig. S6). In “*Ca*. Pseudoramibacter fermentans,” the genes encoding the predicted EtfA and EtfB proteins are not located next to those encoding the ACD enzyme. Instead, the *etfAB* genes are in a cluster with a gene that encodes a homologue of a putative prephenate dehydrogenase (PRDH), an enzyme that catalyzes oxidative decarboxylation in the shikimate pathway for tyrosine biosynthesis (24) (Fig. 3). Pairwise gene expression analysis of transcripts encoding the ACD, PRDH, EtfA, and EtfB proteins (Fig. 7) showed that ACD had strong correlations with EtfA (*r*^2^ = 0.93) and EtfB (*r*^2^ = 0.91), suggesting coregulation of ACD and the electron transport flavoproteins and supporting a role for an electron-bifurcating ACD in the reverse β-oxidation cycle of “*Ca*. Pseudoramibacter fermentans,” which we also predict for “*Ca*. Weimeria bifida.”

The revised genome sequence of “*Ca*. Pseudoramibacter fermentans” also contained complete sets of genes for subunits of the RNF and the Ech complexes, a characteristic also shared with “*Ca*. Weimeria bifida.” The transcript abundances of “*Ca*. Pseudoramibacter fermentans” genes encoding subunits of the RNF complex were at or near the 90th percentile throughout the time series analyzed in this experiment (Fig. 6B), whereas the transcript abundances for the genes predicted to encode the “*Ca*. Pseudoramibacter fermentans” Ech hydrogenase were below the median at most time points after the addition of lignocellulosic biorefinery residues (Fig. 6C). Furthermore, the other hydrogenase predicted to be present in “*Ca*. Pseudoramibacter fermentans,” Hyd1, had transcript abundances that were higher than those of genes encoding the Ech hydrogenase complex. These observations suggest that energy conservation in “*Ca*. Pseudoramibacter fermentans” primarily occurs via generation of an ion motive force by the RNF complex. However, hydrogen production via Hyd1 could be important to maintain redox balance during chain elongation by “*Ca*. Pseudoramibacter fermentans.”

Hydrolysis of the elongated acyl-CoA molecule relies on a CoAT or a thioesterase (Fig. 2). The “*Ca*. Pseudoramibacter fermentans” genome encodes a CoAT and two predicted thioesterases, each of which was expressed at or above median level compared to levels of other transcripts analyzed during this experiment (Fig. 6D). The abundances of the transcripts encoding CoAT and one of the predicted thioesterases (TE.2) (Fig. S6) have higher correlations with those of predicted enzymes in the reverse β-oxidation pathway than the transcripts of the other predicted thioesterase (TE.1) (Fig. S6). As was the case with TE.1 in “*Ca*. Weimeria bifida,” an amino acid sequence analysis of the TE.2 from “*Ca*. Pseudoramibacter fermentans” showed that it belongs to the hotdog fold superfamily of thioesterases. Specifically, the TE.2 “*Ca*. Pseudoramibacter fermentans” thioesterase is predicted to be a PaaI family enzyme, which includes the medium-chain acyl-CoA thioesterase II of Escherichia coli (25). Thus, the transcriptomic analysis suggests that both CoAT and TE.2 may participate in acyl chain release during MCFA synthesis in “*Ca*. Pseudoramibacter fermentans.”

### (v) “*Ca*. Pseudoramibacter fermentans” is predicted to use multiple routes for glycerol metabolism.

**FIG 8:**
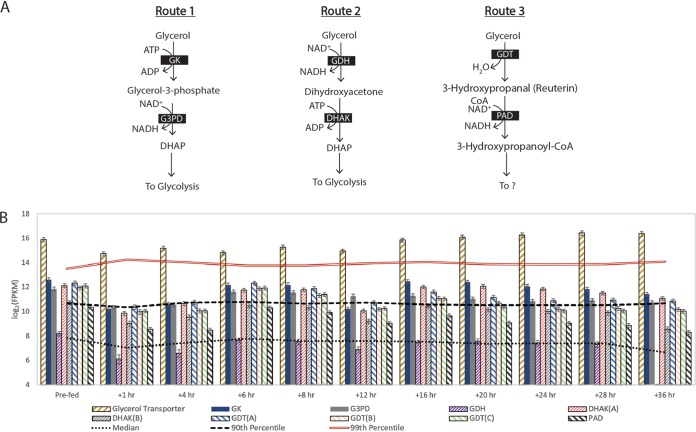
Glycerol utilization by “*Ca*. Pseudoramibacter fermentans.” (A) “*Ca*. Pseudoramibacter fermentans” contains genes for three glycerol utilization routes. Key enzymes in these pathways include glycerol kinase (GK), glycerol-3-phosphate dehydrogenase (G3PD), glycerol dehydrogenase (GDH), dihydroxyacetone kinase (DHAK), glycerol dehydratase (GDT), and propionaldehyde dehydrogenase (PAD). (B) Transcript abundance for glycerol transport and utilization. Subunits of a single enzyme complex are indicated in parentheses. “Prefed” represents the time point prior to feeding the reactor. Error bars show 95% confidence levels determined with CuffLinks.

Glycerol is known to be a significant carbon source in the lignocellulosic biorefinery residues used in this and earlier studies (8). Analysis of the reactor medium showed that all of the glycerol was removed within the first 6 h (Fig. S2), suggesting that it is a favored carbon source for one or more of the microbes in this microbiome. The gene most highly expressed by “*Ca*. Pseudoramibacter fermentans” at several time points after the addition of lignocellulosic biorefinery residues encodes a predicted glycerol transporter (Fig. 8B), suggesting that glycerol is rapidly transported by this organism.

To assess how “*Ca*. Pseudoramibacter fermentans” metabolizes glycerol, we monitored transcript abundances of genes predicted to be involved in this process. The revised “*Ca*. Pseudoramibacter fermentans” genome also predicts that this organism contains enzymes to metabolize glycerol, and the time series transcriptomics data showed that this organism expressed genes for three putative glycerol conversion pathways (Fig. 8). The first route predicted to be active in “*Ca*. Pseudoramibacter fermentans” (Fig. 8A, route 1) uses an ATP-dependent glycerol kinase, in which the resultant glycerol-phosphate is oxidized to produce dihydroxy-acetone phosphate (DHAP). The second predicted route in “*Ca*. Pseudoramibacter fermentans” (Fig. 8A, route 2) uses glycerol dehydrogenase to oxidize glycerol to dihydroxyacetone before it is phosphorylated to DHAP. In a third predicted route in “*Ca*. Pseudoramibacter fermentans” (Fig. 8A, route 3), glycerol is converted to 3-hydroxypropanal (reuterin), a compound proposed to inhibit growth of other microbes by inducing oxidative stress (26). All of the above predicted glycerol utilization genes, with the exception of glycerol dehydrogenase, were expressed above median levels throughout the 36-h time series (Fig. 8B). Thus, it is possible that multiple, or even all three, pathways (Fig. 8A) play a role in glycerol metabolism in “*Ca*. Pseudoramibacter fermentans.”

**FIG 9:**
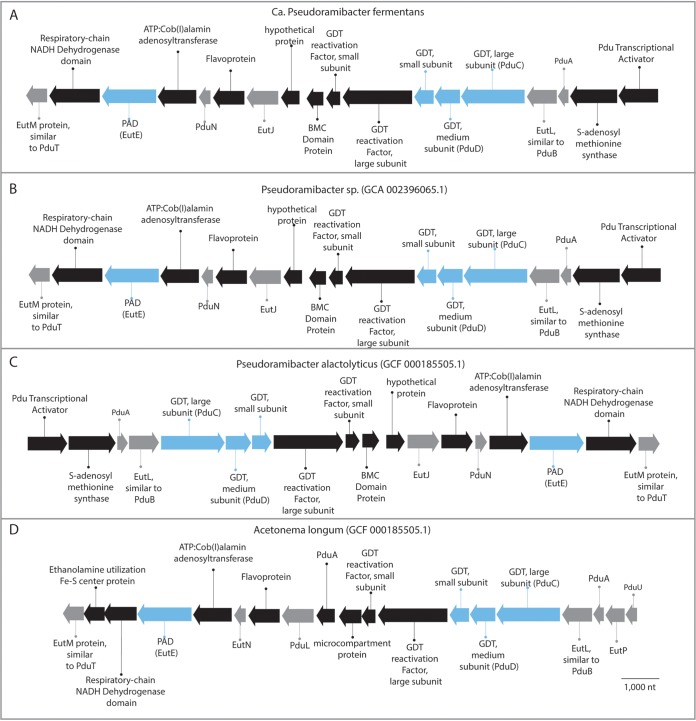
Glycerol utilization gene clusters in “*Ca*. Pseudoramibacter fermentans” (A), related *Pseudoramibacter* species (B and C), and Acetonema longum (D). Blue shading indicates a gene predicted to encode an enzyme involved in route 3 (Fig. 8A), and gray shading indicates a gene predicted to encode a microcompartment protein.

In addition to expressing genes encoding pathways for multiple glycerol utilization routes, “*Ca*. Pseudoramibacter fermentans” contains a gene cluster encoding predicted polyhedral body proteins involved in propanediol utilization along with genes encoding the multiple subunits of a glycerol dehydratase (Fig. 9). Thus, it is possible that these predicted protein microcompartments may help protect “*Ca*. Pseudoramibacter fermentans” from a toxin like reuterin or from other toxins that are potential products of glycerol metabolism by one or more routes in this organism.

Overall, our data provide new evidence that “*Ca*. Pseudoramibacter fermentans” directs glycerol to central carbon metabolism and potentially produces reuterin, a toxic compound that may impact the growth and abundance of other members in this microbiome. One possible outcome of glycerol metabolism to intermediates that can be further transformed via glycolysis (Fig. 8) is the conversion of glycerol to MCFA. A free-energy analysis of glycerol conversion to butyrate, hexanoate, and octanoate shows that all of these reactions are energetically feasible when hydrogen is produced to maintain internal redox balance (Table 2).

**TABLE 2:** Thermodynamics of glycerol conversion to butyrate, hexanoate, and octanoate^*a*^

Description reaction	Overall reaction	Δ*G*^0^′ per mol glycerol (kJ mol^−1^)
Conversion to butyrate	2 C_3_H_8_O_3_ → 1 C_4_H_7_O_2_^−^ + 2 CO_2_ + 1 H^+^ + 4 H_2_	−188
Conversion to hexanoate	3 C_3_H_8_O_3_ → 1 C_6_H_11_O_2_^−^ + 3 CO_2_ + 1 H_2_O + 1 H^+^ + 5 H_2_	−306
Conversion to octanoate	4 C_3_H_8_O_3_ → 1 C_8_H_15_O_2_^−^ + 4 CO_2_ + 2 H_2_O + 1 H^+^ + 6 H_2_	−423

a Free energies of formation used to calculate Δ*G*^o^′ are provided in Table S2 in the supplemental material.

### (vi) “*Ca*. Pseudoramibacter fermentans” is predicted to use an electron-confurcating lactate dehydrogenase for MCFA production.

**FIG 10:**
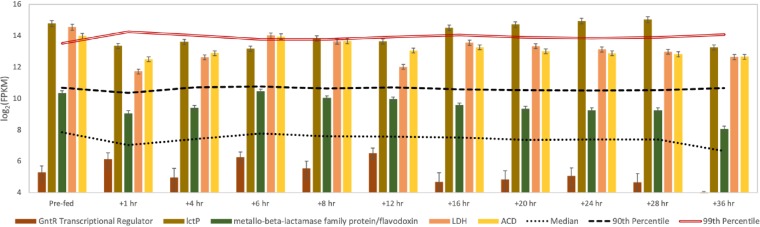
Transcript abundance for a lactate utilization gene cluster in “*Ca*. Pseudoramibacter fermentans.” Genes include a transcriptional regulator, lactate permease (*lctP*), a potential flavodoxin, lactate dehydrogenase (LDH), and acetyl-CoA dehydrogenase (ACD). “Prefed” represents the time point prior to feeding the reactor. Error bars show 95% confidence levels determined with CuffLinks.

We previously proposed that one member of this MCFA-producing microbiome consumes lactate produced by other community members (8). The “*Ca*. Pseudoramibacter fermentans” genome assembled in this study contains a gene cluster encoding a lactate transporter (lactate permease), lactate dehydrogenase (LDH), and ACD (Fig. 3J). All three of these genes are expressed above the 90th percentile at all time points after the addition of lignocellulosic biorefinery residue (Fig. 10), supporting their role in MCFA production from lactate.

**FIG 11:**
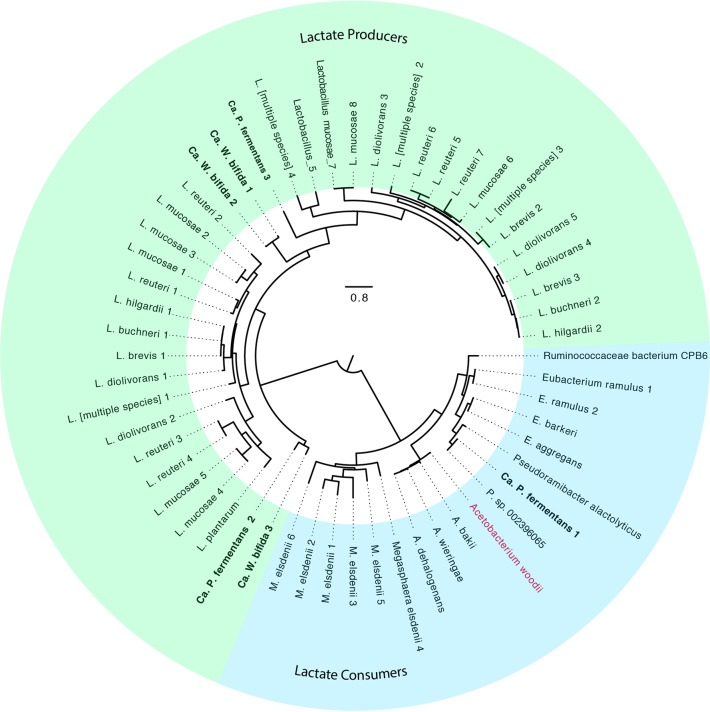
A maximum-likelihood phylogenetic tree of lactate dehydrogenase amino acid sequences among different members of the *Firmicutes* phylum. Anaerobic lactate consumers contain lactate dehydrogenase and form a distinct cluster from lactate producers, such as those found among the lactobacilli.

The amino acid sequence of the predicted “*Ca*. Pseudoramibacter fermentans” LDH in this gene cluster is most closely related to predicted flavin adenine dinucleotide (FAD)-binding oxidoreductase amino acid sequences from several related organisms, including other *Firmicutes* and organisms in the *Fusobacterium* phylum (Data File S8). The “*Ca*. Pseudoramibacter fermentans” LDH clusters with amino acid sequences of proteins from organisms that consume lactate anaerobically (Fig. 11), including two bacteria known to convert lactate to MCFA, *Ruminococcaceae* CPB6 and M. elsdenii (5, 7), and with the LDH from Acetobacterium woodii that uses electron confurcation to couple lactate and ferredoxin oxidation with NAD^+^ reduction to overcome the thermodynamic bottleneck of lactate oxidation (27). Pairwise correlation analyses of transcript abundance (Fig. 7) for the “*Ca*. Pseudoramibacter fermentans” LDH with EtfA (*r*^2^ = 0.77) and EtfB (*r*^2^ = 0.65) suggest a role for this dehydrogenase and the electron transfer flavoproteins in anaerobic lactate oxidation. Therefore, we hypothesize that in “*Ca*. Pseudoramibacter fermentans,” the electron transfer flavoproteins EtfA and EtfB have a role both in electron bifurcation by ACD in the reverse β-oxidation cycle and in electron confurcation with LDH.

### (vii) Predicted conservation of metabolic functions between “*Ca*. Pseudoramibacter fermentans” and related organisms.

The organization of genes encoding the complete reverse β-oxidation pathway in “*Ca*. Pseudoramibacter fermentans” is similar to that found in the other two genomes available for *Pseudoramibacter* organisms (Fig. 3). In all three cases, the gene encoding ECoAH clusters with other genes in the reverse β-oxidation pathway, while the *etfAB* genes are found in a separate region of the genome. In all three organisms, the reverse β-oxidation genes are located near the genes encoding fatty acid biosynthesis (Fig. 3). While other investigators have suggested a potential role for fatty acid biosynthesis genes in MCFA production (28), our data do not provide support for this hypothesis since transcripts for genes encoding enzymes involved in reverse β-oxidation were orders of magnitude more abundant than fatty acid biosynthesis genes (Fig. S7).

The ability of “*Ca*. Pseudoramibacter fermentans” to metabolize glycerol appears to be common in the *Pseudoramibacter* genus as genes for the three proposed routes of glycerol metabolism in “*Ca*. Pseudoramibacter fermentans” are also found in the other two *Pseudoramibacter* genomes. This is also the case for the presence of genes predicted to produce protein microcompartments in a cluster with genes encoding glycerol dehydratase (Fig. 9). The production of polyhedral protein microcompartments thought to encase a diol dehydratase when 1,2-propanediol is consumed has been described in other organisms, such as Salmonella enterica (a gammaproteobacterium) and Acetonema longum (Fig. 9D), a member of the *Negativicutes* class within the *Firmicutes* phylum (29, 30). In addition to the two other *Pseudoramibacter* genomes which contain glycerol gene clusters identical to those of “*Ca*. Pseudoramibacter fermentans” (Fig. 9B and C), a similar glycerol utilization cluster was identified in A. longum (Fig. 9D).

### Concluding remarks.

Our results reveal several previously unexplored metabolic and energetic features of chain-elongating bacteria. Multi-omic analysis of “*Ca*. Weimeria bifida” suggests that this organism may have a previously undescribed ability to use both the pentose phosphate and the phosphoketolase pathways for pentose consumption and production of acetyl-CoA that is needed for MCFA synthesis. Further, both “*Ca*. Weimeria bifida” and “*Ca*. Pseudoramibacter fermentans” may use multiple hydrogenases, including a proton-translocating energy-conserving hydrogenase (EchABCDEF), to support MCFA production. Although both chain elongators contained genes for the RNF complex, the transcriptomic evidence suggests that this complex may be more important for generating an ion motive force in “*Ca*. Weimeria bifida.” Our data also predict that “*Ca*. Pseudoramibacter fermentans” uses several routes to consume glycerol as a carbon source, with potentially toxic intermediates sequestered in protein microcompartments, and a thermodynamic analysis supports the ability of “*Ca*. Pseudoramibacter fermentans” to produce MCFA from this substrate. Finally, our data implicate an electron-confurcating LDH in providing carbon skeletons needed to support MCFA production from lactate by “*Ca*. Pseudoramibacter fermentans.” Further work is necessary to elucidate the implications of the genomic features uncovered in this study on the bioenergetics of MCFA production, to assess whether similar processes are involved in the production of MCFA from other substrates, and to develop a better understanding of microbial pathways for production of additional valuable products from renewable organic materials.

### Summary descriptions of novel organisms.

The formal proposals of the new *Candidatus* genus “*Candidatus* Weimeria” and new species “*Candidatus* Weimeria bifida” and “*Candidatus* Pseudoramibacter fermentans” are given in Table 3.

**TABLE 3:** Protologues for “*Ca*. Weimeria bifida” and “*Ca*. Pseudoramibacter fermentans”

Characteristic or parameter	Value for “*Candidatus* Weimeria bifida”	Value for “*Candidatus* Pseudoramibacter fermentans”
Taxon no.	NA^*a*^	NA
Species name	“*Candidatus* Weimeria bifida”	“*Candidatus* Pseudoramibacter fermentans”
Genus name	“*Candidatus* Weimeria”	*Pseudoramibacter*
Specific epithet	bifida	fermentans
Genus etymology	Wei.mer’ia. N.L. fem. n., named after Paul J. Weimer, a pioneer in using rumen microbes to produce valuable chemicals	Pseu.do.ra.mi.bac’ter. Gr. adj. *pseudês*, false; L. masc. n. *ramus*, a branch; N.L. masc. n. *bacter*, rod, staff; N.L. masc. n. *Pseudoramibacter*, false branching rod
Type species of the genus	“*Candidatus* Weimeria bifida”	Pseudoramibacter alactolyticus (GCA_000185505.1)
Taxon number of the type species	NA	NA
Genus status	gen. nov.	Existing, validly published name
Species etymology	bi’fi.da. L. fem. adj*. bifida*, divided into two parts, as in the bifid shunt	fer.men’tans. L. part. adj*. fermentans*, fermenting
Species status	sp. nov.	sp. nov.
Designation of the type MAG	VOGC00000000	VOGB00000000
MAG/SAG accession number	VOGC01000000	VOGB01000000
Genome status	Draft	Draft
Genome size (bp)	2,394,983	2,285,558
GC mol%	45.9	50.2
Country of origin	USA	USA
Region of origin	Wisconsin	Wisconsin
Source of sample	Bioreactor	Bioreactor
Sampling date(s)	Multiple	Multiple
Geographic location	University of Wisconsin—Madison	University of Wisconsin—Madison
Latitude	43.035801 N	43.035801 N
Longitude	89.359081 W	89.359081 W
Depth	NA	NA
Altitude	NA	NA
Temperature of the sample (°C)	35	35
pH of the sample	5.5	5.5
Relationship to O_2_	Anaerobe	Anaerobe
Energy metabolism	Heterotroph	Heterotroph
Assembly (no. of samples)	5	5
Sequencing technology	Illumina HiSeq 2000 and PacBio	Illumina HiSeq 2000 and PacBio
Binning software used	Anvio, version 5	Anvio, version 5
Assembly software used	Spades, version 3.12	Spades, version 3.12
Habitat	Anaerobic bioreactor	Anaerobic bioreactor
Miscellaneous, extraordinary features relevant for the description	When a bioreactor community, seeded from acid digester sludge collected at the Nine Springs Wastewater Treatment Plant (Madison, WI), is fed lignocellulosic biorefinery residues, “*Ca*. Weimeria bifida” is predicted to use the phosphoketolase pathway (bifid shunt) to degrade xylose and produce butyrate and medium-chain fatty acids via reverse β-oxidation. “*Ca*. Weimeria bifida” is predicted to maintain redox balance by using a ferredoxin-dependent hydrogenase or an energy-conserving Ech hydrogenase to produce hydrogen. While other organisms within the proposed “*Ca*. Weimeria” genus contain genes for pentose degradation via the oxidative pentose phosphate pathway, “*Ca*. Weimeria bifida” is unique in containing genes for the bifid shunt.	When lignocellulosic biorefinery residues are fed to a bioreactor community that was seeded from acid digester sludge collected at the Nine Springs Wastewater Treatment Plant (Madison, WI), “*Ca.* Pseudoramibacter fermentans” is predicted to produce medium-chain fatty acids via reverse β-oxidation, using lactate and glycerol as the organic substrates. “*Ca.* Pseudoramibacter fermentans” contains genes for multiple glycerol utilization routes, including conversion of glycerol to reuterin. Lactate utilization is predicted to involve an electron-confurcating lactate dehydrogenase. In addition, an electron-bifurcating acyl-CoA dehydrogenase is predicted to be used for energy conservation, with reduced ferredoxin produced by this enzyme used by the RNF complex for the creation of an ion motive force to support ATP production. “*Ca.* Pseudoramibacter fermentans” is predicted to produce hydrogen via a ferredoxin-dependent hydrogenase as a mechanism for balancing internal redox conditions.

a NA, not available.

## MATERIALS AND METHODS

### Bioreactor operation.

We operated a bioreactor containing 150 ml of liquid. Lignocellulosic biorefinery residues, prepared as described previously (2), were added into the bioreactor, and reactor liquid was pumped out of the bioreactor every hour to maintain a residence time in the reactor of 6 days. The pH of the reactor was controlled at 5.5 by adding 5 M KOH through a pump attached to a pH controller. The temperature of the bioreactor was maintained at 35°C using a water bath. For the 36-h time series experiment, 28 ml of liquid was removed from the reactor, and 28 ml of lignocellulosic biorefinery residues was added one time, bringing the starting liquid volume to 150 ml.

### Reactor sampling.

Prior to feeding the reactor with 28 ml of lignocellulosic biorefinery residues, samples were collected for metagenomic and metatranscriptomic analyses. Samples for DNA sequence analysis were collected in 2-ml centrifuge tubes and centrifuged at 10,000 × *g* for 10 min. After the supernatant was decanted, cell pellets were stored at –80°C until DNA was extracted. Samples for RNA were collected in 2-ml centrifuge tubes and centrifuged at 10,000 × *g* for 1.5 min. After the supernatant was decanted, samples for RNA were flash frozen in liquid nitrogen and then stored at –80°C until RNA was extracted. Three samples for RNA extraction and sequencing were collected as a control prior to adding the conversion residue. At each time point after the addition of lignocellulosic biorefinery residues, one sample was collected for RNA extraction, and the supernatant from this sample was used for high-performance liquid chromatography (HPLC) and gas chromatography mass spectrometry (GC-MS) analyses, as described previously (2).

### DNA sequencing.

DNA was extracted from biomass samples using a phenol-chloroform extraction method described previously (8). For all metagenomic samples (days 96, 120, 168, 252, and 378), sequencing was performed with an Illumina HiSeq 2500 sequencer to generate 2- by 250-bp reads. For the day 378 sample, PacBio sequencing and library preparation were performed by the Joint Genome Institute. The sequence library was prepared using a PacBio 10-kb low-input library preparation, and libraries were sequenced with a PacBio Sequel.

### Metagenomic analysis.

Quality checking and trimming of Illumina DNA reads were performed using the program Sickle (31). The resulting reads from days 96, 120, 168, 252, and 378 were coassembled using metaSPAdes, version 3.12.00 (32). The assembled contigs were binned using Anvio, version 5 (33). After binning, draft genomes were gap filled with the PacBio reads from day 378 using PBSuite, version 15.8.24 (34). Quality checking was performed on the gap-filled draft genomes using CheckM (35). Draft genomes were annotated with MetaPathways, version 2.5 (36). Taxonomic assignments based on single-copy marker genes were made with the GTDB tool kit (9), and phylogenetic trees were constructed using RAxML (37). Average nucleotide identities were calculated with JSpecies using the ANIb algorithm (38).

### Metatranscriptomic analysis.

RNA was extracted, and cDNA was synthesized and sequenced as described previously (8). RNA was collected for 11 time points starting on day 252 of reactor operations: before feeding the conversion residue to the bioreactor (“prefed”) and at various time points for 36 h after feeding (+1, +4, +6, +8, +12, +16, +20, +24, +28, and +36 h). Triplicate samples were taken for the prefed time point and underwent separate extraction, rRNA depletion, cDNA synthesis, library preparation, and sequencing. Quality checking and trimming of raw reads were performed with Sickle (31), and rRNA reads were removed with SortMeRNA (39). Reads from each time point were mapped to open reading frames in the MAGs with CuffLinks (40). Confidence intervals (95%) were calculated using CuffLinks using the triplicate sample for the prefed time point to estimate variance for each transcript.

### Data availability.

The whole-genome shotgun project has been deposited at DDBJ/ENA/GenBank under the accession numbers VOGB00000000 and VOGC00000000. The versions described in this paper are versions VOGB01000000 and VOGC01000000. The raw DNA sequences are available in the National Center for Biotechnology Information Sequence Read Archive (SRA) database under BioProject PRJNA418244 for days 96 and 120 and BioProject PRJNA535528 for days 168, 252, and 378. The raw RNA sequences are available in the SRA database under BioProject PRJNA535528.

## Supplementary Material

Supplemental file 1

Supplemental file 2

Supplemental file 3

Supplemental file 4

Supplemental file 5

Supplemental file 6

Supplemental file 7

Supplemental file 8

Supplemental file 9
