# Supplementary material for: Medium-Chain Fatty Acid Synthesis by “Candidatus Weimeria bifida” gen. nov., sp. nov., and “Candidatus Pseudoramibacter fermentans” sp. nov
Source: Appl Environ Microbiol. 2020 Jan 21;86(3):e02242-19. doi: 10.1128/AEM.02242-19 (PMC6974650; doi:10.1128/AEM.02242-19)
Supplement: Supplemental file 1 [file zam003209562s1.pdf]

SUPPLEMENTAL MATERIAL FOR:

Medium-chain fatty acid synthesis by *Candidatus* Weimeria bifida, gen. nov., sp. nov., and *Candidatus* Pseudoramibacter fermentans, sp. nov.

Matthew J. Scarborough[1,2], Kevin S. Myers[1], Timothy J. Donohue[1,3], and Daniel R. Noguera[1,2]*

1. The Great Lakes Bioenergy Research Center, UW-Madison, Madison, WI
2. The Department of Civil and Environmental Engineering, UW-Madison, Madison, WI
3. The Department of Bacteriology, UW-Madison, Madison, WI

*Corresponding author e-mail: dnoguera@wisc.edu

Table S1. Summary of metagenome-assembled genomes from MCFA-producing bioreactor

MAG Name	Original Name[1]	Relative Abundance[2] (%)	Completeness (%)[3]	Contamination (%)[3]	Genome size (Mbp)[3]	No. scaffolds[3]
Ca. W. bifida (LCO1.1)	LCO1	75.3	96.9 [95.4]	0.5 [0.0]	2.39 [2.10]	10 [44]
Ca. P. fermentans (EUB1.1)	EUB1	4.7	99.2 [97.8]	0.2 [0.2]	2.29 [2.00]	29 [35]
COR1.1	COR1	2.4	95.0 [99.2]	6.7 [0.8]	2.41 [2.51]	82 [225]
COR3.1	COR3	2.8	98.4 [98.4]	2.4 [7.4]	3.02 [3.65]	134 [533]
COR4.1[4]		1.1	100	0.7	2.45	8
LAC1.1	LAC1	3.8	99.5 [99.5]	1.1 [1.1]	2.77 [2.63]	9 [18]
LAC2.1	LAC2	2.0	99.4 [99.4]	1.6 [1.6]	3.18 [3.18]	37 [79]
LAC4.1	LAC4	1.6	97.7 [98.9]	0.6 [1.3]	3.14 [3.35]	53 [95]
LAC5.1	LAC5	2.5	99.2 [80.1]	0.0 [0.8]	2.11 [1.48]	6 [181]
LAC6.1[4]		1.9	99.1	1.1	2.80	12
LAC7.1[4]		2.0	99.1	2.8	3.41	33

[1] Names reported in Scarborough et al. (1)
[2] Relative abundance based in DNA read mapping normalized to genome size for day 252 sample.
[3] Numbers in brackets indicate values for assembled MAGs reported in Scarborough et al. (1)
[4] MAGs not assembled in Scarborough et al. (1)

Table S2. Free energy of formation values used for thermodynamic calculations.[1]

Compound Name	Formula	ΔG_f (kJ mol^{-1})
Glycerol	$C_3H_8O_3$	-486.09
Butyrate	$C_4H_7O_2^-$	-349.40
Hexanoate	$C_6H_{11}O_2^-$	-335.85
Octanoate	$C_8H_{15}O_2^-$	-322.29
Water	H_2O	-237.17
Carbon dioxide (aq)	CO_2	-386.02
Hydrogen ion (pH = 7)	H^+	-39.87
Hydrogen (Gas)	H_2	0

[1]Free energies of formation (ΔG_f^0) are from Thauer et al. 1977 (2). For octanoate, similar to the assumption made by Thauer et al. for hexanoate, each CH_2 group decreases the free energy of formation by 8.32 kJ/mol in the butyrate to octanoate series.

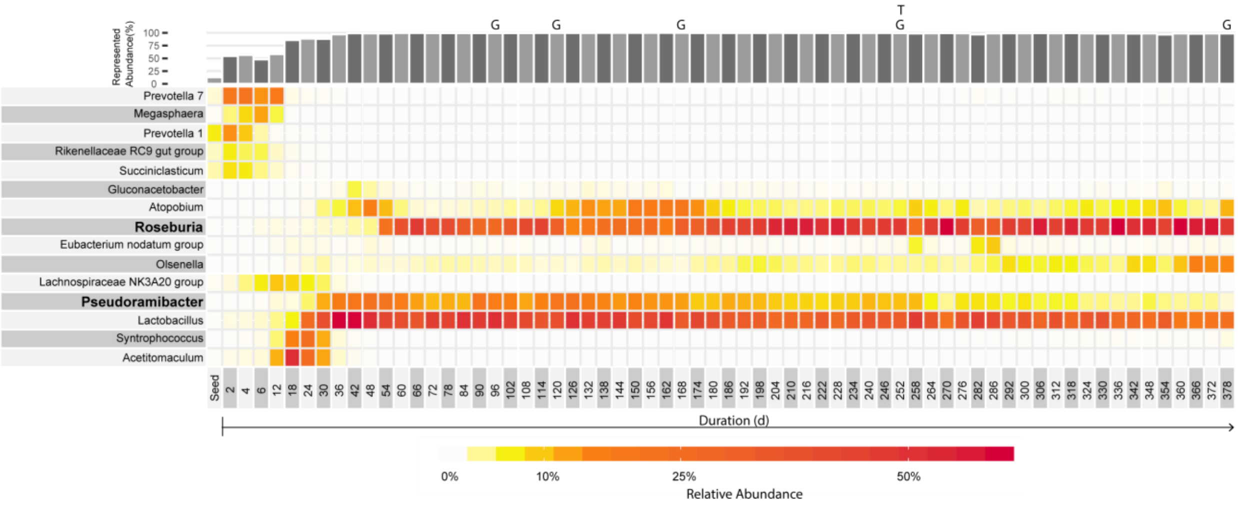

Figure S1. Relative abundance of bacteria in the bioreactor based on 16S rRNA gene amplicon sequencing. With the short 16S rRNA gene fragments, *Ca.* W. bifida was classified as a member of the *Roseburia* genus, whereas *Ca.* P. fermentans was correctly classified as a member of the *Pseudoramibacter* genus. The first column shows results from the acid digester sludge ("seed") used for reactor inoculum. The duration after starting the bioreactor is shown on the x-axis and genera names are provided on the y-axis. The bar plot above the heatmap shows the sum of abundance represented in the heatmap. Colors in the heatmap indicate relative abundance with higher abundance indicated by red color intensity. Samples corresponding to metagenomic and metatranscriptomic samples analyzed in this study are shown with "G" indicating a metagenomic sample and "T" indicating the time point used for the time-series metatranscriptomic analysis. The 16S-based abundance for the first 252 days was previously published in Scarborough et al. 2018 (3).

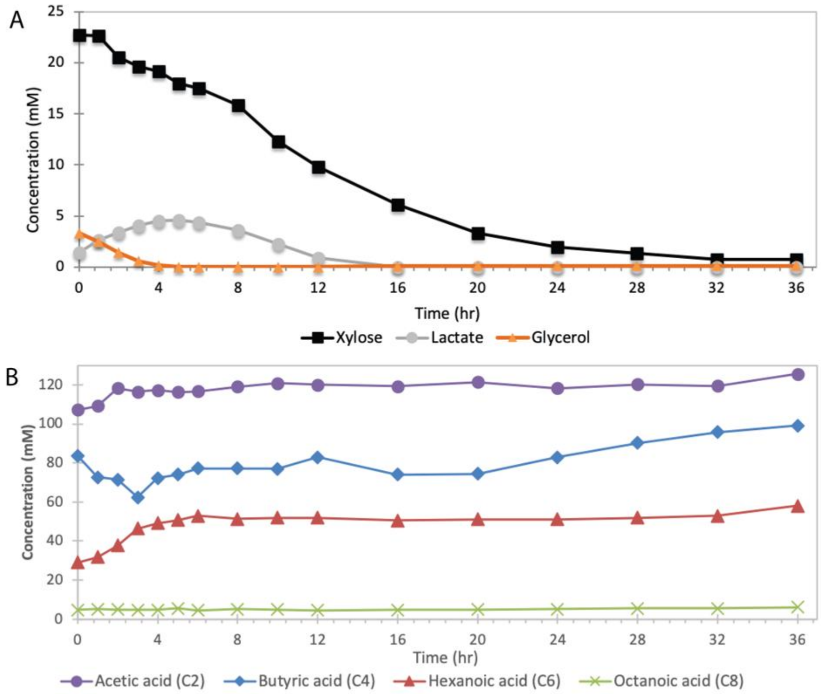

Figure S2. Extracellular metabolites for 36 hours after addition of lignocellulosic biorefinery residues. (A) Xylose, lactate, and glycerol are all compounds that are consumed during the course of the experiment. (B) The reactor produced various carboxylic acid end products, including acetic acid, butyric acid, hexanoic acid, and octanoic acid.

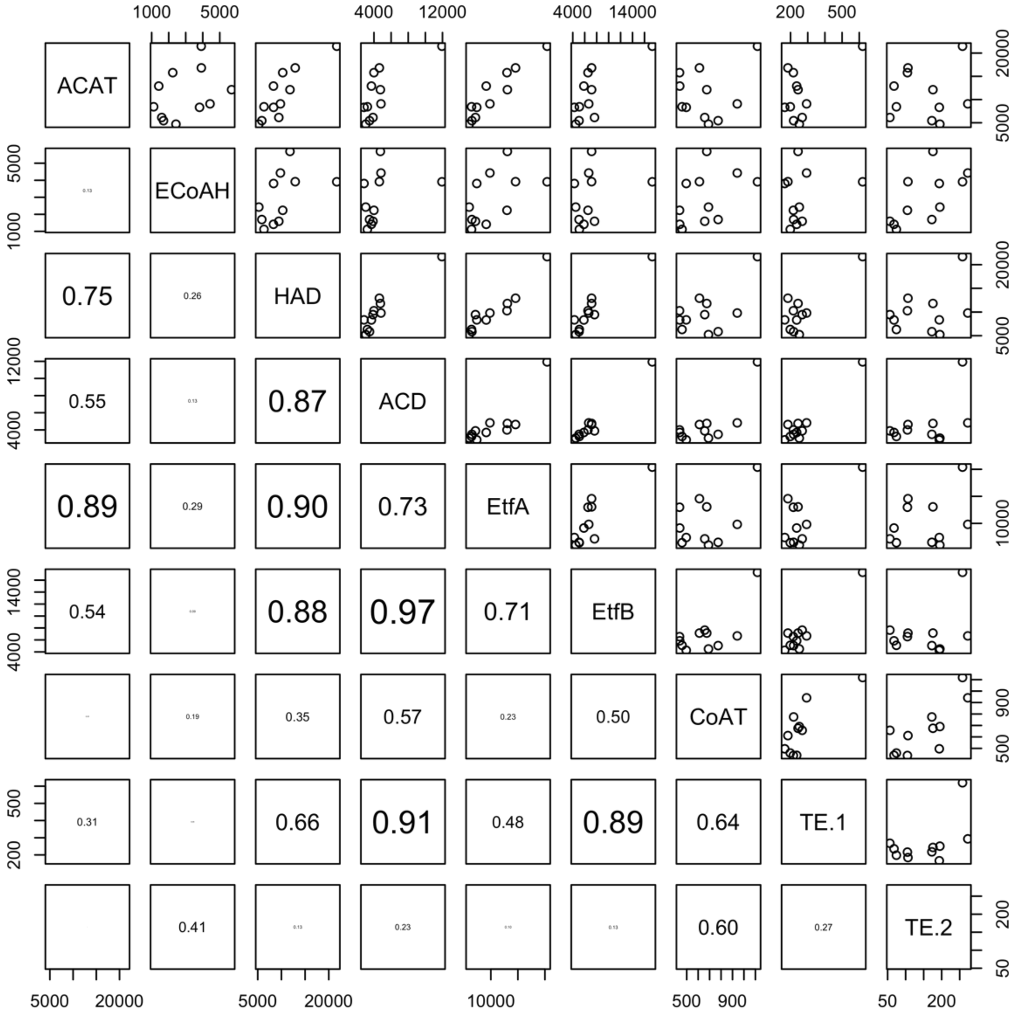

Figure S3. Pairwise comparison of transcript abundance for reverse β-oxidation genes in *Ca.* W. bifida. Coefficients of determination are shown in bottom-left panels.

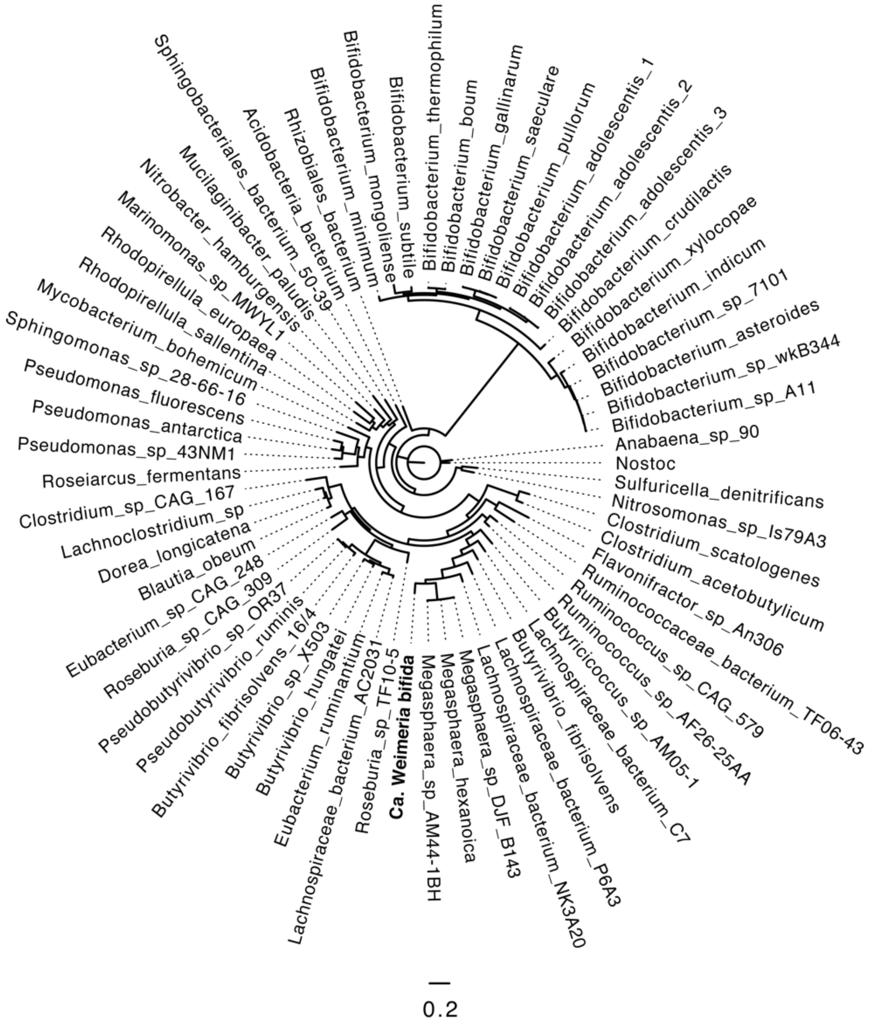

Figure S4. A maximum-likelihood phylogenetic tree of the phosphoketolase enzyme across several phyla of bacteria. Sequences across several phyla were selected and show a distinct cluster of the amino acid sequences for *Bifidobacterium* species. The sequence from *Ca.* W. bifida clustered with those from members of the chain-elongating genus *Megasphaera*.

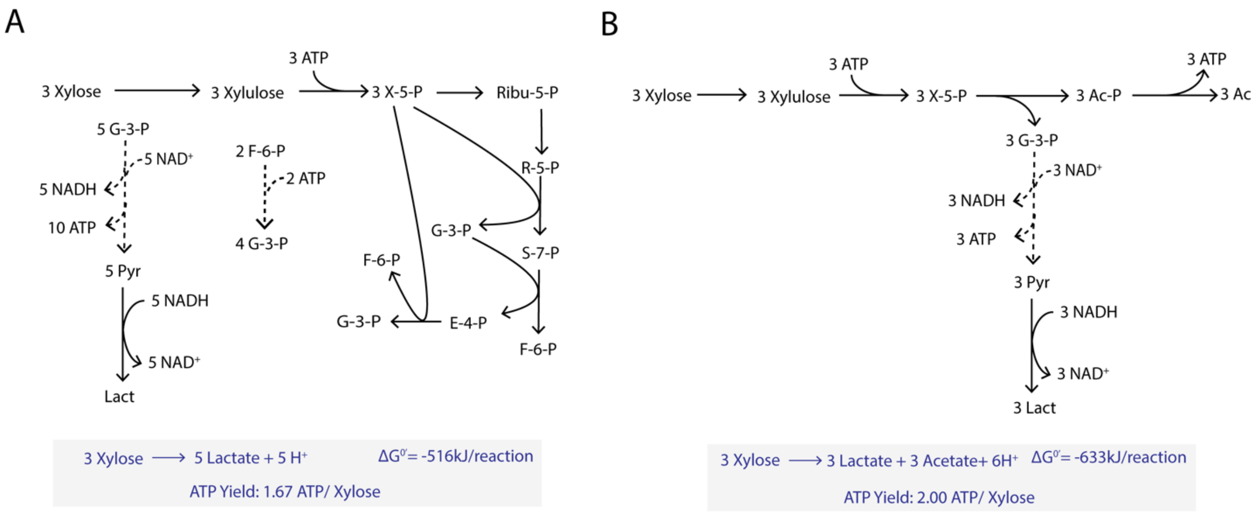

Figure S5. Comparison of ATP yields and thermodynamics with (A) consumption of xylose via the pentose phosphate pathway and (B) homolactic acid fermentation with the pentose phosphate pathway. Both pathways are described in Tanaka et al. (4).

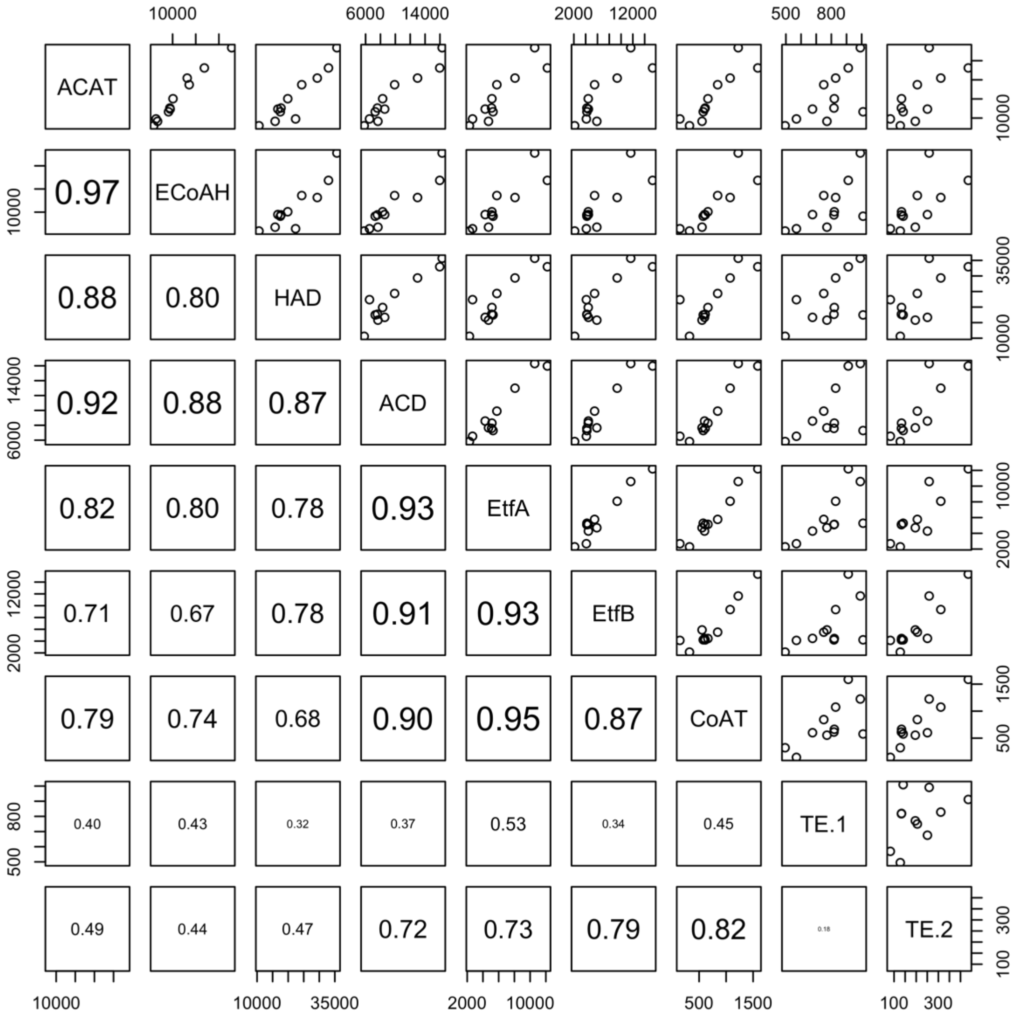

Figure S6. Pairwise comparison of transcript abundance for reverse β-oxidation genes in *Ca.* P. fermentans. Coefficients of determination are shown in bottom-left panels.

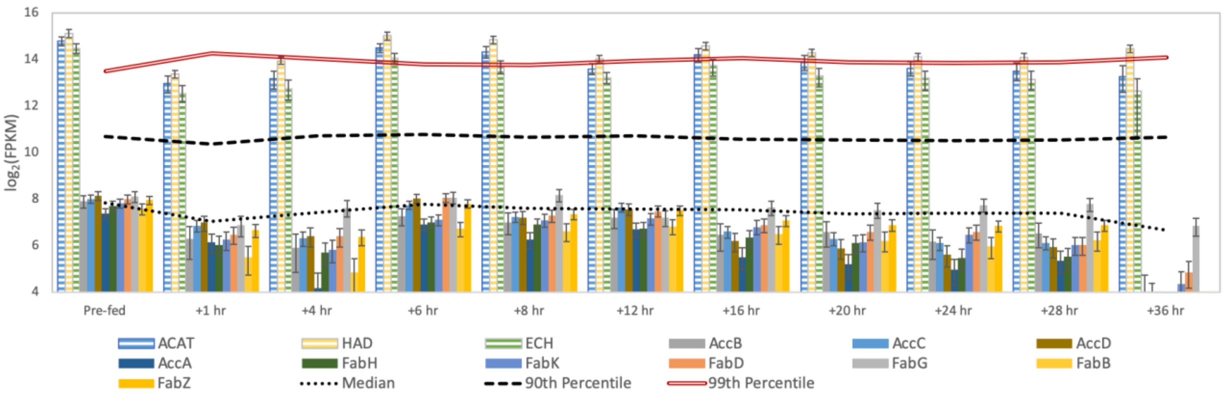

Figure S7. Abundance of reverse β-oxidation genes and fatty acid biosynthesis transcripts for *Ca.* P. fermentans. The genes are ordered as they are predicted to be ordered in the *Ca.* P. fermentans genome (**Fig. 3J**).

REFERENCES

1. Scarborough MJ, Lawson CE, Hamilton JJ, Donohue TJ, Noguera DR. 2018. Metatranscriptomic and thermodynamic insights into medium-chain fatty acid production using an anaerobic microbiome. mSystems doi:0.1128/msystems.00221-18. 0.1128/msystems.00221-18.

2. Thauer RK, Jungermann K, Decker K. 1977. Energy conservation in chemotrophic anaerobic bacteria. Bacteriological Reviews 41:100-180.

3. Scarborough MJ, Lynch G, Dickson M, McGee M, Donohue TJ, Noguera DR. 2018. Increasing the economic value of lignocellulosic stillage through medium-chain fatty acid production. Biotechnology for Biofuels 11:200. 10.1186/s13068-018-1193-x.

4. Tanaka K, Komiyama A, Sonomoto K, Ishizaki A, Hall SJ, Stanbury R. 2002. Two different pathways for D-xylose metabolism and the effect of xylose concentration on the yield coefficient of L-lactate in mixed-acid fermentation by the lactic acid bacterium Lactococcus lactis 10-1. Applied Microbiology and Biotechnology 60:160-167. 10.1007/s00253-002-1078-5.
